# Supplementary material for: Abnormal thrombosis and neutrophil activation increase hospital-acquired sacral pressure injuries and morbidity in COVID-19 patients
Source: Front Immunol. 2023 Mar 21;14:1031336. doi: 10.3389/fimmu.2023.1031336 (PMC10070761; doi:10.3389/fimmu.2023.1031336)
Supplement: Supplementary file 1 [file DataSheet_1.pdf]

Supplementary Material

Supplementary Table 1: Clinical and demographic characteristics of hospitalized patients during the study period.

		COVID (+)	COVID (-)	p-value[a]
Total Admissions		5,482	53,284	
Total Hospitalization Days		64,980	526,783	
Total HASPIs		49	244	
HASPI Rate		7.5 per 10,000 days	4.6 per 10,000 days	
Pressure Ulcer Risk at Admission[b]		19 (15-20)	19 (16-21)	**<0.001**
Female Sex		2,635 (47.6%)	26,620 (49.7%)	**0.003**
Age (Years)		69.4 (58.8-79.8)	66.3 (53.5-76.7)	**<0.001**
Race	White	3,513 (63.5%)	37,962 (70.9%)	**<0.001**
	Black	1,623 (29.3%)	12,654 (23.6%)	
	Other	395 (7.1%)	2,912 (5.4%)	
Hospitalization Length (Days)		8 (6-14)	7 (6-11)	**<0.001**
ICU Admission: Yes		2,210 (40.0%)	18,656 (34.9%)	**<0.001**
Intubation: Yes		104 (1.9%)	602 (1.1%)	**<0.001**

Bolded values denote statistically significant p-values.

[a]Based on Wilcoxon-rank sum test for continuous variable comparisons and Chi-squared test for categorical variable comparisons.

[b]Based on Braden risk assessment score closest to admission. Note that a lower score equates to greater risk for ulcer formation.

Supplementary Table 2: Comparison of hospital course in COVID-19 (+) patients with and without HASPIs.

		HASPI (+)	HASPI (-)	p-value[a]
**N**		**49**	**5,482**	**--**
Pressure Ulcer Risk at Admission[b]		14 (12-16.5)	19 (15-20)	**<0.001**
Female Sex		17 (34.7%)	2,618 (47.8%)	0.08
Age (Years)		66.3 (56.7-74.9)	69.4 (58.9-79.8)	0.22
Race	White	29 (59.2%)	3,484 (63.6%)	0.20
	Black	19 (38.8%)	1,604 (29.3%)	
	Other	1 (2.0%)	394 (7.2%)	
Hospitalization Length (Days)		29 (19.5-35)	8 (6-14)	**<0.001**
Co-morbidities	Hypertension	31 (63.3%)	3,230 (58.9%)	0.53
	COPD	13 (26.5%)	976 (17.8%)	0.11
	Diabetes	23 (46.9%)	1,912 (34.9%)	0.08
Hospital Course Severity	Hospitalization Only	4 (8.2%)	3,306 (60.3%)	**<0.001**
	ICU	25 (51.0%)	1,456 (26.6%)	
	Ventilation	9 (18.4%)	65 (1.2%)	
	Death	11 (22.5%)	655 (22.5%)	

Bolded values denote statistically significant p-values.

[a]Based on Wilcoxon-rank sum test for continuous variable comparisons and Chi-squared or Fisher's Exact tests for categorical variables comparisons.

[b]Based on Braden risk assessment score closest to admission.

Supplementary Table 3: Multivariable ordinal regression for worsening hospital course severity in COVID-19 (+) patients.

Outcome	Regression Estimate(95% CI)	p-value[a]
HASPI[b]: Yes vs. No	2.2 (1.8-2.8)	**<0.001**
Age (Per additional year)	0.99 (0.99-1.00)	0.42
Hypertension: Yes vs. No	0.93 (0.88-0.99)	**0.01**
COPD[c]: Yes vs. No	1.00 (0.99-1.1)	0.11
Diabetes: Yes vs. No	1.1 (1.0-1.2)	**<0.001**

Bolded values denote statistically significant p-values.

[a]These p-values represent multivariable ordinal regression analysis controlling for age and patient co-morbidities (hypertension, chronic obstructive pulmonary disease, congestive heart failure, and diabetes) with disease severity as the outcome.

[b]HASPI: Hospital-acquired sacral pressure injury.

[c]COPD: Chronic obstructive pulmonary disease.

Supplementary Table 4: Thirty-day HASPI-related morbidity.

	COVID (+)	COVID (-)	p-value[a]
**N**	**47**	**247**	
30-day morbidity[b]	8 (16.3%)	28 (11.5%)	0.34
Debridement: Yes	7 (14.3%)	14 (5.7%)	**0.047**
HASPI Infection: Yes	4 (8.2%)	20 (8.2%)	0.99

Bolded values denote statistically significant p-values.

[a]Based on Fisher's Exact tests.

[b]Morbidity is defined as an aggregate of either requiring surgical debridement of HASPI or infection of HASPI from time of admission to 30-days after hospital discharge.

Supplementary Table 5: ICD-10 codes used to search for healthcare acquired sacral pressure injury.

L89.150, L89.151, L89.152, L89.153, L89.154, L89.159 - pressure ulcer of sacral region (various stages)
L98.429- non-pressure ulcer of sacrum
L98.499- non-pressure chronic ulcer of skin
L89.30, L89.31, L89.32, L89.302- pressure ulcer of buttocks
L98.411, L98.499- non-pressure ulcer of buttocks
L89.322, L89.323, L89.324, L89.312, L89.313, L89.314, L89.156, L89.90- pressure ulcer of left buttock (stages 2,3,4, or unspecified), right buttock (stages 2,3,4, or unspecified)

Supplementary Figure Legends

Supplementary Figure 1. Diagram showing the recruitment process.

(A) Among the 143,527 patients hospitalized at our institution during our defined study period (March 2020-December 2020), 58,766 patients met our inclusion/exclusion criteria and comprised our study cohort. A total of 3,352 patients had a diagnosis of sacral ulcer during their hospitalization with or without an image of their sacral region in their electronic medical records. Of these patients, 2,992 had their sacral ulcers documented present on admission (POA) and 67 were seen at an outside hospital and were excluded from our study. Our final study cohort consisted of 293 patients with HASPIs in the COVID-19(+) and COVID-19(-) groups.

Supplementary Figure 2. Enlarged photomicrographs of patients described in Figure 1. (A) Biopsy from the patient described in Figure 1A (400x) reveals intraluminal thrombi; occasional neutrophils (*black arrows*) and leukocytoclasis (*grey arrows*) can be seen. **(B)** A photomicrograph (400x) from a biopsy of the patient descried in Figure 1B reveals a rare vascular thrombosis (*black arrow*). Other vessels display intact neutrophils within their lumens (*grey arrow*). **(C)** A photomicrograph (400x) from a biopsy of the patient described in Figure 1C shows a mixed inflammatory infiltrate surrounding vessels that are either congested or contain fibrin thrombi (*black arrow*). Neutrophils are intact, without obvious lekocytoclasis. An eosinophil is present within a vascular lumen (*grey arrow*).

Supplementary Figure 3. PTAH/Fibrin and CD61-stained biopsy tissue shown in Figure 1. (A) Biopsy from the patient described in Figure 1A demonstrates numerous intraluminal thrombi with PTAH/Fibrin staining (200x), as well as aggregation of CD61+ platelets (*brown*; 400x) within numerous vessel lumina, consistent with formation of intraluminal thrombi. **(B)** Biopsy of the patient described in Figure 1B reveals mostly patent vessels without evidence of fibrin thrombi with PTAH/Fibrin staining (200x) or aggregation of platelets with CD61 immunohistochemistry (*brown*; 400x); rare CD61+ platelets flowing through vessels are seen. **(C)** Biopsy of the patient described in Figure 1C is negative for intraluminal fibrin deposition (PTAH/Fibrin; 200x); CD61 immunohistochemistry shows platelet aggregation in some vessels, while others are patent *(brown*; 400x).

Supplementary Figure 4. CD61-stained sections of biopsies described in Figure 1. (A) Biopsy from the patient described in Figure 1A reveals the aggregation of platelets (brown) within numerous

vessels, consistent with formation of intraluminal thrombi. **(B)** Biopsy of the patient described in Figure 1B reveals mostly patent vessels without aggregation of platelets; rare platelets flowing through vessels are seen at higher power (see Supplemental Figure 3B). **(C)** Biopsy of the patient described in Figure 1C show platelet aggregation (*brown*) in some vessels, while others are patent.

Supplementary Figure 5. SARS-CoV-2 transcript levels in sacral ulcer biopsies. Sub-analysis of a SARS-CoV-2 probe set included in the Nanostrang panel. Mean±SEM of normalized probe counts graphed. Significance determined by 2 way ANOVA, ****$p<0.0001$. Of note, these differences in transcript level do not persist as significant when false discovery rate (FDR) testing is performed on the entire Nanostring panel dataset.
