# Supplementary figures and images for: Abnormal thrombosis and neutrophil activation increase hospital-acquired sacral pressure injuries and morbidity in COVID-19 patients
Source: Front Immunol. 2023 Mar 21;14:1031336. doi: 10.3389/fimmu.2023.1031336 (PMC10070761; doi:10.3389/fimmu.2023.1031336)

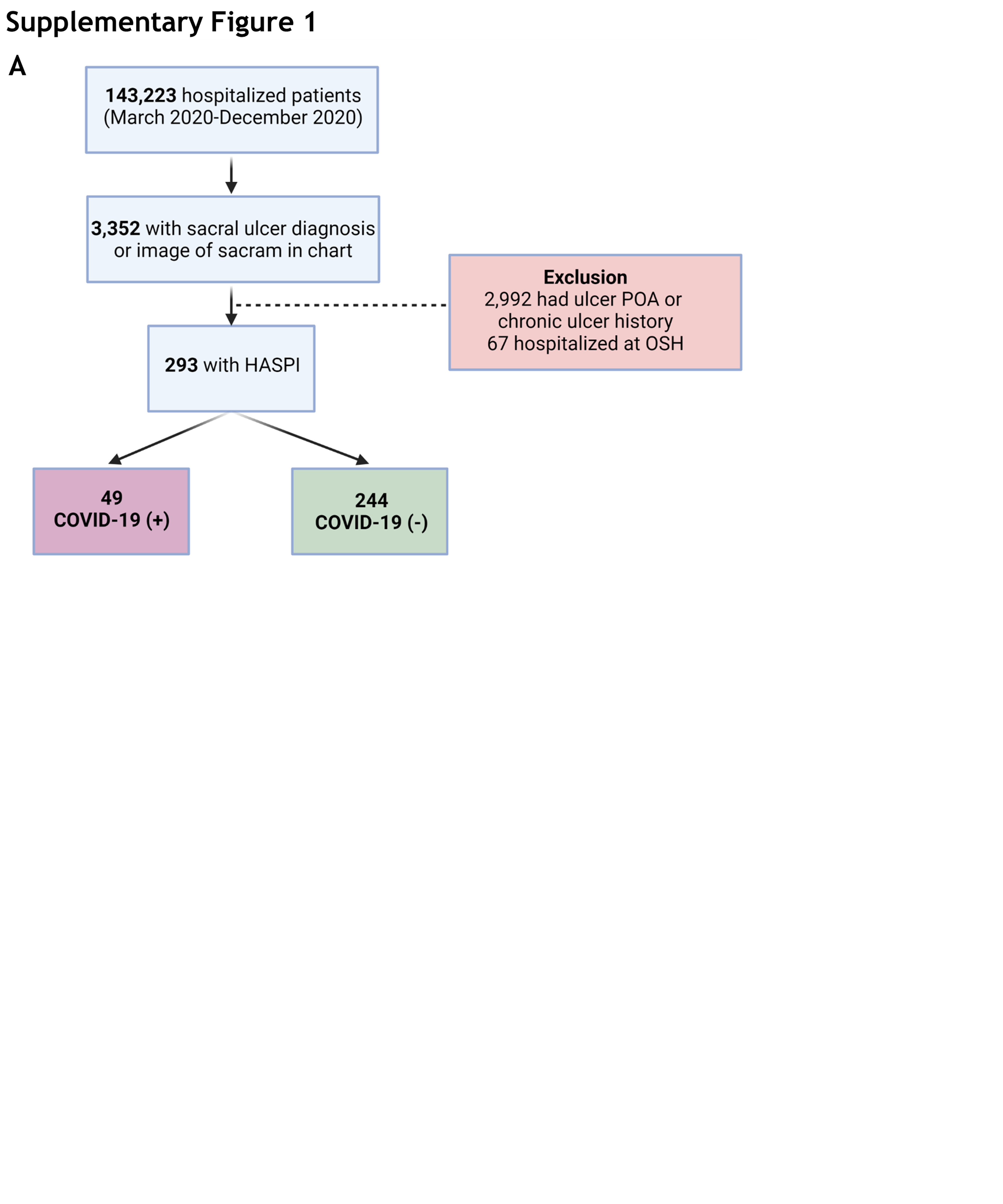

Supplement: Supplementary file 2 [file Image_1.tif]

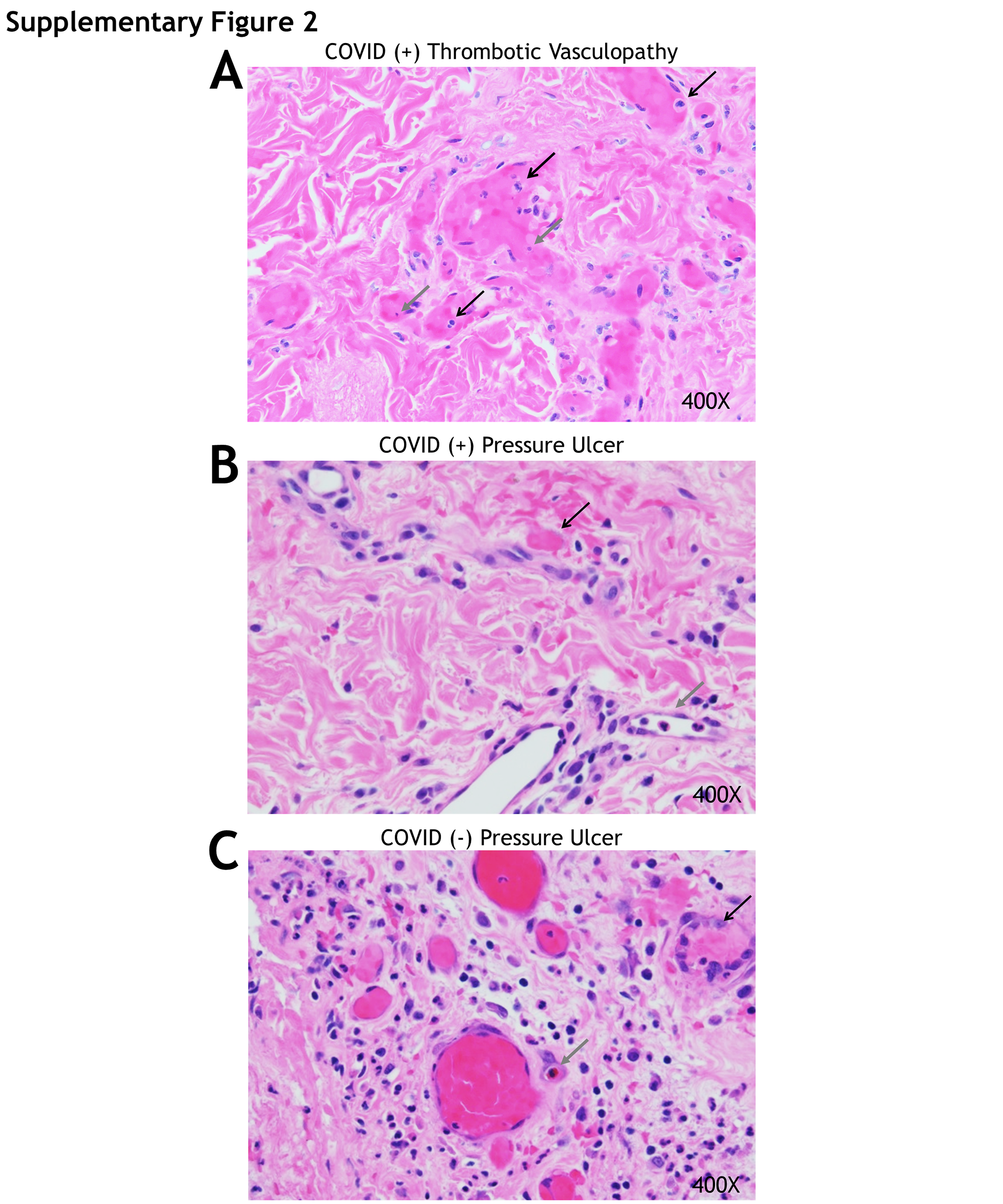

Supplement: Supplementary file 3 [file Image_2.tif]

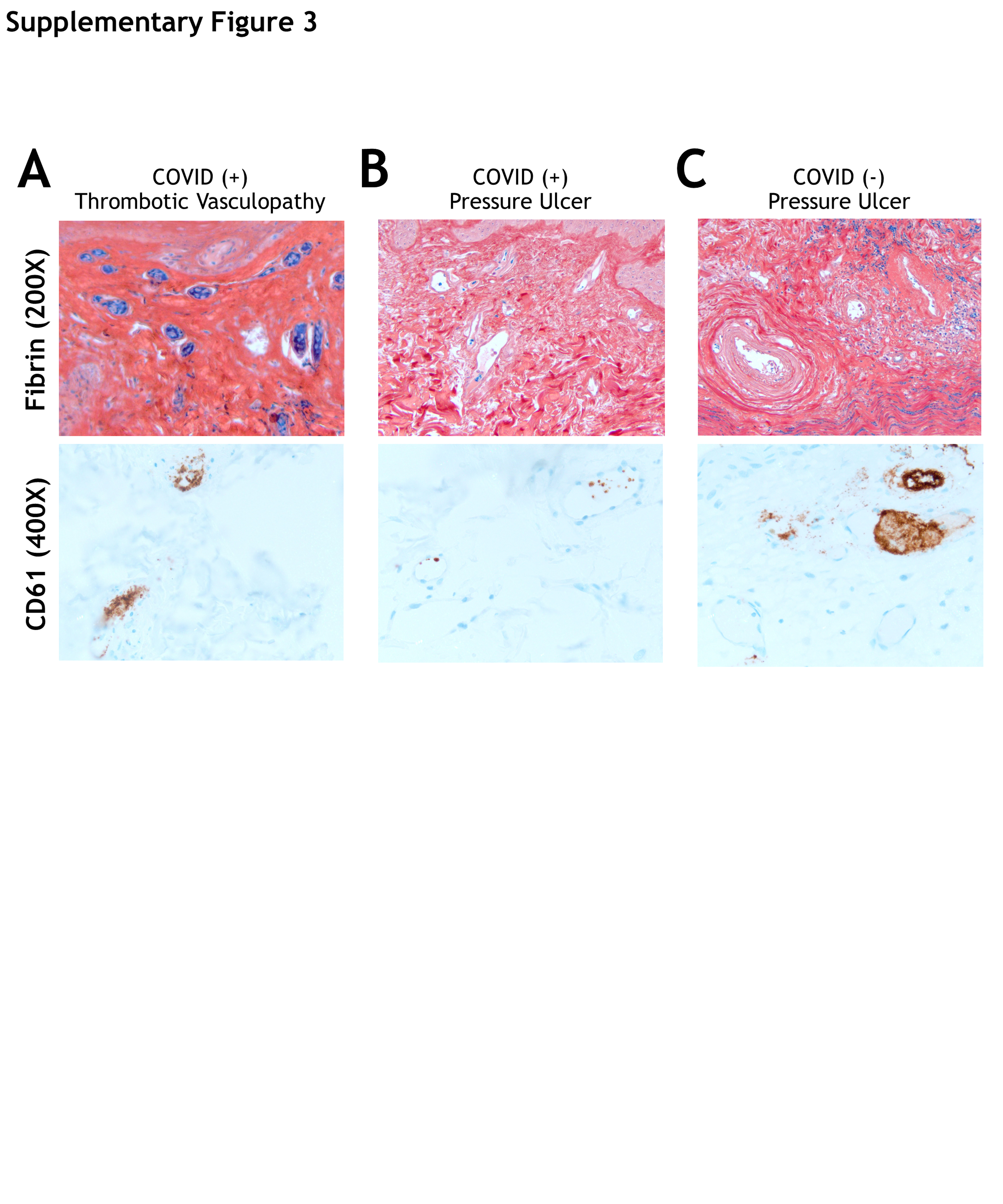

Supplement: Supplementary file 4 [file Image_3.tif]

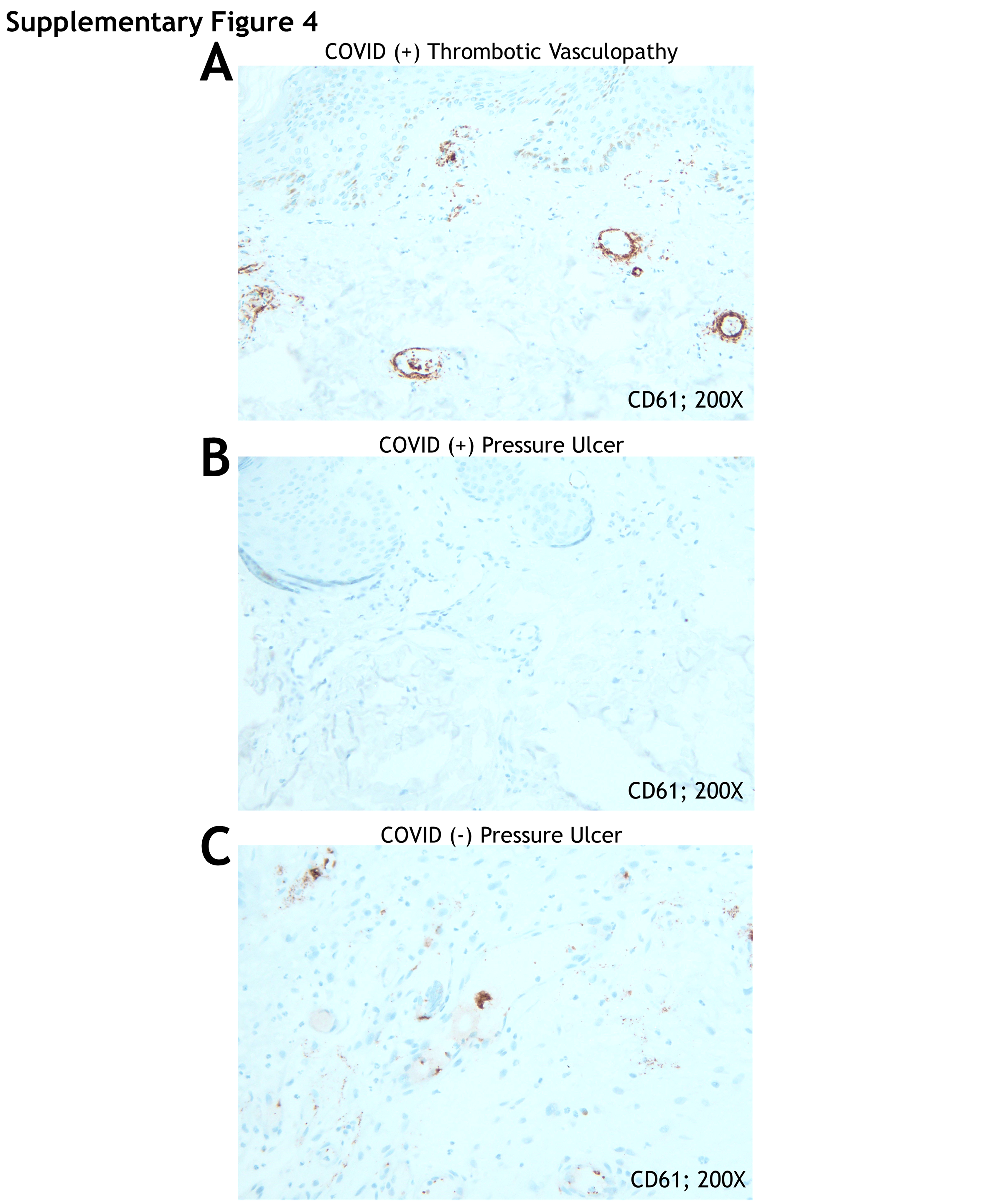

Supplement: Supplementary file 5 [file Image_4.tif]

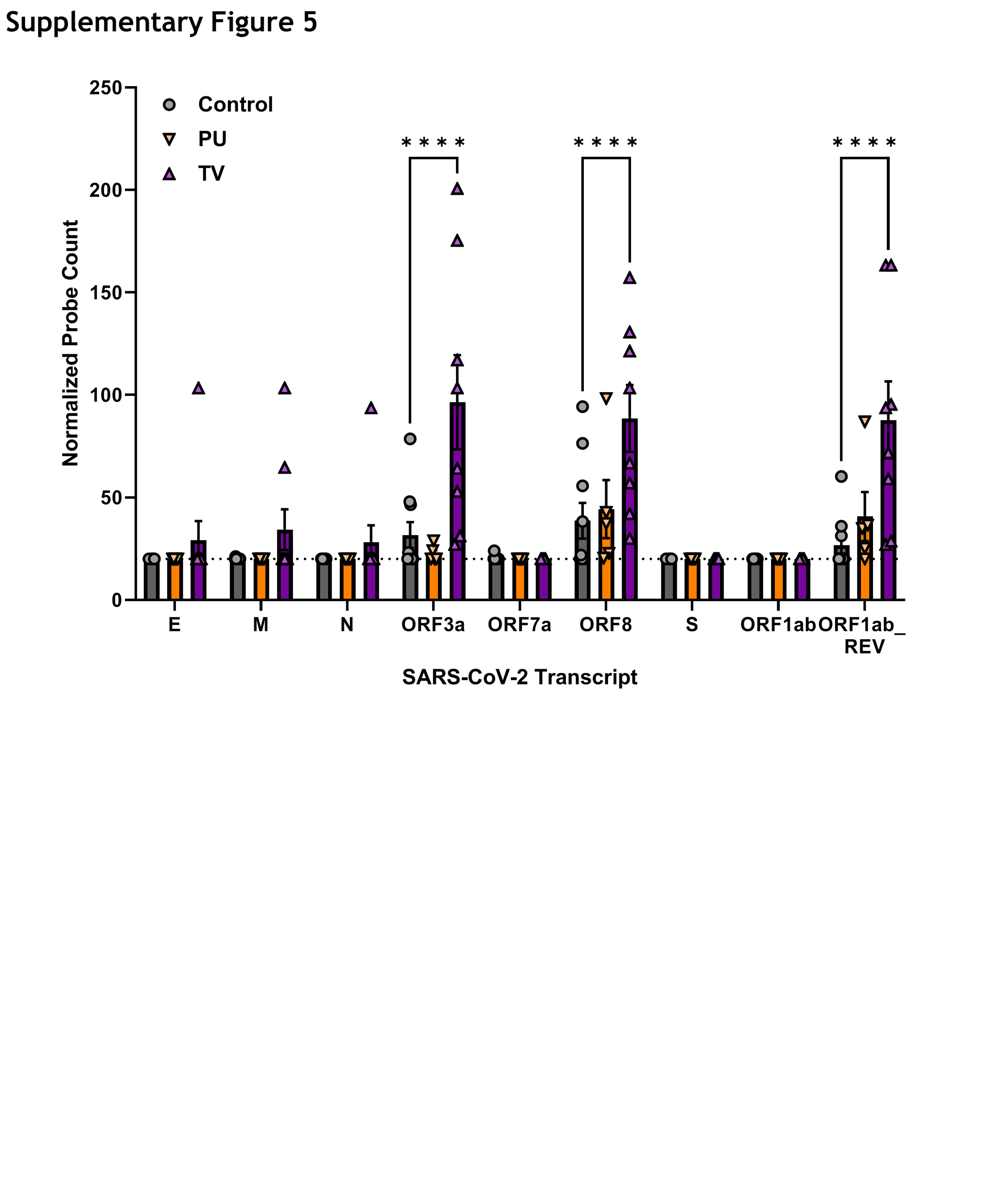

Supplement: Supplementary file 6 [file Image_5.tif]
